# The Early Effect of Carotid Artery Stenting on Antioxidant Capacity and Oxidative Stress in Patients with Carotid Artery Stenosis

**DOI:** 10.1155/2016/1789596

**Published:** 2016-12-13

**Authors:** Slawomir Michalak, Wojciech Ambrosius, Ewa Wysocka, Mieczyslaw Dziarmaga, Robert Juszkat, Andrzej Wykretowicz, Wojciech Kozubski

**Affiliations:** ^1^Department of Neurochemistry and Neuropathology, Poznan University of Medical Sciences, Przybyszewskiego Str. 49, 60-355 Poznan, Poland; ^2^Department of Neurology, Poznan University of Medical Sciences, Przybyszewskiego Str. 49, 60-355 Poznan, Poland; ^3^Chair and Department of Laboratory Diagnostics, Poznan University of Medical Sciences, Szamarzewskiego Str. 82/84, 60-569 Poznan, Poland; ^4^Department of Cardiology-Intensive Therapy, Poznan University of Medical Sciences, Przybyszewskiego Str. 49, 60-355 Poznan, Poland; ^5^Department of General and Interventional Radiology, Poznan University of Medical Sciences, Dluga Str. 1/2, 61-848 Poznan, Poland

## Abstract

The treatment of carotid artery stenosis is associated with the risk of complications, which may include stroke after carotid artery stenting (CAS) and myocardial infarction after carotid endarterectomy (CEA). The imbalance between prooxidative mechanisms and antioxidant capacity creates a milieu of factors, which may increase the risk of complications after endovascular procedures. We have examined 43 consecutive patients with carotid artery stenosis. Sera were analyzed for the activity of paraoxonase (PON) and arylesterase (ARE), sulfhydryl groups (SG), malondialdehyde (MDA), and conjugated dienes (CD) concentrations by means of spectrophotometric methods before and next day after CAS. We have found lowered PON (*P* = 0.0032), increase in ARE activity (*P* = 0.0058), and decrease in sulfhydryl groups concentration (*P* = 0.0267). No effect on absolute MDA and CD concentrations was observed. The degree of carotid artery stenosis correlated negatively with PON/ARE ratio after CAS (*r*_*S*_ = −0.507, *P* = 0.0268). To conclude, CAS influences both enzymatic (differently, PON and ARE activity) and nonenzymatic antioxidant defense. Females are more susceptible to lipid peroxidation after CAS. PON/ARE ratio after CAS correlated with the degree of carotid artery stenosis. The changes (deltas) in ARE activity, SG, and MDA concentrations correlated with the severity of neurological deficit and disability.

## 1. Introduction

The stenosis of carotid artery is one of the well identified risk factors of ischemic stroke [1]. Carotid endarterectomy (CEA) is a surgical procedure used in patients with carotid stenosis to reduce ischemic stroke risk [2]. Over the last decade carotid artery stenting (CAS) became an alternative method for the treatment of carotid stenosis. The most severe complications of vascular interventions include stroke, myocardial infarction, or fatal event. No differences in the incidence of those complications after CEA and CAS were found in long-term observation; however in the periprocedural period, stroke risk was higher after CAS, while risk of myocardial infarction was higher after CEA [3]. On the other hand, recently, no differences were observed in early and later periprocedural complications after both CEA and CAS [4].

Oxidative stress and the balance between prooxidative mechanisms and antioxidant capacity create a milieu of factors, which may increase the risk of complications after endovascular procedures. Oxidative stress recognized as an imbalance between production of toxic reactive species ((ROS) reactive oxygen species and RNS (reactive nitrogen species)) and antioxidative defense may be initiated by ischemia, reperfusion and/or mechanical injury.

The generation of ROS during early reperfusion can cause important lesions in ischemic organs, and, in the brain, it leads to blood-brain barrier breakdown, cerebral edema, haemorrhagic transformation, and neuronal death [5].

The effects of mechanical action during vascular interventions include vascular smooth muscle cells lesion as an effect of apoptosis [6], inflammation [7], and cell proliferation [6]. In experimental balloon model short-term effect of mechanical injury to the artery was smooth muscle cells apoptosis caused by the mechanisms involving oxidative stress [8], which was observed already 90 minutes after endovascular procedure. ROS can be generated by either vascular smooth muscle cells [9] or endothelial cells [10].

Thus, the effectiveness of antioxidant systems can play an important role in the protection against complications of endovascular procedures.

Paraoxonases 1 and 3 (PON1 and PON3) are enzymes localized in high-density lipoproteins (HDL) [11], while paraoxonase 2 (PON2) is not linked to HDL [12]. PON is a class A, calcium dependent esterase [13], which protects low-density lipoproteins (LDL) against oxidation [11, 12]. Arylesterase (ARE), an isoenzyme of PON, is a carboxylesterase that catalyzes splitting of aromatic esters of fatty acids [14] and shows specific activity against phenyl acetate [15]. PON and ARE belong to enzymatic antioxidant system in plasma and hydrolytic reactions catalyzed by the enzymes, which are crucial for protection against peroxidation of plasma lipids. In our previous study we have identified PON and ARE activities and PON/ARE ratio as predictors of the outcome after ischemic stroke [16].

Sulfhydryl groups (SG), which represent a majority of thiols, play a crucial role in nonenzymatic plasma antioxidant systems. Proteins, albumins especially, are the principal source of SG in plasma [17]. The concentration of plasma sulfhydryl groups decreases during acute cerebral ischemia and correlates with immune response in stroke patients [18].

Prooxidative processes produce a number of molecules which may indicate the intensity of oxidative stress. Oxidation of fatty acids leads to generation of conjugated dienes (CD), which reflects plasma lipids peroxidation. The accumulation of conjugated dienes was observed in cases of ischemia followed by reperfusion of brain tissue [19]. Malondialdehyde (MDA) is a product of lipids peroxidation. Numerous studies reported increased serum MDA levels in stroke patients and their effect on the outcome [20–23].

We are not aware of the studies on paraoxonase/arylesterase complex enzymatic antioxidant defense after carotid artery stenting. The aim of this study was to estimate the effect of CAS on PON and ARE activities, SG level, and CD and MDA concentrations in the very early phase after the intervention.

## 2. Materials and Methods

### 2.1. Patients

The study included 43 consecutive patients with carotid artery stenosis (31 males, 12 females) aged 64 ± 9 years admitted to the Department of Cardiology-Intensive Therapy, Poznan University of Medical Sciences. Carotid artery stenosis was diagnosed based on ultrasound examination performed twice. All patients enrolled in the study had carotid stenosis ≥ 70%. Carotid artery stenting was performed with the use of routine procedure and self-expanding nitinol stents. We have excluded from the study patients who underwent endovascular or surgical procedures over last 6 months. The study protocol was accepted by Ethic Committee of Poznan University of Medical Sciences.

### 2.2. Laboratory Methods

Whole blood samples were taken before CAS and in the morning besides endovascular intervention from fasting patients. Serum was isolated after centrifugation and stored at −80°C until time of analysis.

The activities of paraoxonase (PON) and arylesterase (ARE) were estimated spectrophotometrically with the use of paraoxon and phenyl acetate as respective substrates according to previously described methods [24, 25].

Malondialdehyde (MDA) concentration was analyzed based on thiobarbituric acid reaction [26].

Conjugated dienes (CD) concentrations were estimated spectrophotometrically by means of Recknagel and Glende Jr. method [27] and calculated using the coefficient of molar absorbance (*ε* = 2,8 × 10^4^/M × cm).

Sulfhydryl groups (SG) concentrations were evaluated spectrophotometrically based on the reaction with 5,5′-dithiobis (2-nitrobenzoic acid) (DTNB) according to Ellman's method [28].

The routine laboratory tests performed included hematology tests, lipid profile (total cholesterol [T-C], high-density lipoprotein cholesterol [HDL-C], low-density lipoprotein cholesterol [LDL-C], and triacylglycerols [TAG]), and glucose levels before carotid angioplasty.

### 2.3. Clinical Examinations and Definition of Outcomes

Neurological examination including medical history was performed at admission according to the study protocol. Patients neurological deficit, disability, and activities of daily living were measured using National Institute of Health Stroke Scale (NIHSS), modified Rankin scale, and Barthel index. Clinimetric assessment was performed after the patient was included in the study and in the morning besides endovascular intervention by study neurologist.

Hypertension was defined as a history of treated hypertension, with ischemic heart disease as a history of myocardial infarction, angina, coronary artery bypass grafting, or percutaneous coronary intervention [29]. Patients were defined as diabetic if their fasting glucose level was ≥7.00 mmol/L (126 mg/dL) at least twice within an interval of at least 48 hours or if they were taking antidiabetic medication.

### 2.4. Statistics

Statistical analysis was performed with the use of licensed MedCalc software. First, we have tested the distribution of results with D'Agostino-Pearson test. Then, results with Gaussian distribution were analyzed with Student's *t*-test for paired samples or independent variables and non-Gaussian distribution with nonparametric Wilcoxon or Mann-Whitney test depending on the type of variable. The correlations between PON, ARE activities, MDA, CD and SG concentrations and clinical data, clinimetric measures, and routine laboratory tests were tested with the use rank correlation test. Logistic regression was used for the testing of the change (difference between values after and before carotid angioplasty) in PON, ARE activities, MDA, CD, and SG concentrations in the models including routine laboratory tests and clinical data.

## 3. Results

Demographic and clinical data, medical history, and comorbidities in the studied cohort of patients are presented in Table 1. No differences in age, carotid stenosis, clinimetric scores, and medical history between female and male patients were found (Table 1). One-third of patients underwent vascular procedures previously: coronary artery bypass grafting (CABG) (33%), percutaneous coronary intervention (PCI) (25%), carotid artery stenting (contralateral to current qualification) (20%), endarterectomy (10%), aortoiliac prosthesis (10%), and femoral artery revascularization (10%).

The results of routine laboratory analyses performed before CAS are presented in Table 2. No severe abnormalities were observed; namely, the general status of all patients was stable.

**Figure 1:**
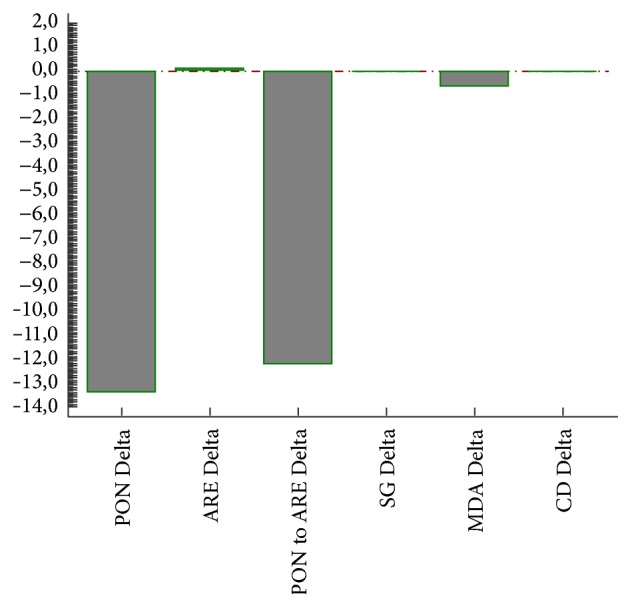
The differences (deltas) in PON and ARE expressed in U/L, SG in *μ*M/L, MDA in nM/L, and CD in *μ*M/L in all patients enrolled in the study.

Carotid artery stenting caused lowering in PON activity, increase in ARE activity, and decrease in sulfhydryl groups concentration (Table 3). No effect on absolute MDA and CD concentrations was observed. In Figure 1 the changes (deltas) in analyzed parameters after CAS are presented. Females are more susceptible to the increase in lipid peroxidation after CAS than males (Table 4), when the change (delta) in analyzed parameters was taken into consideration.

**Figure 2:**
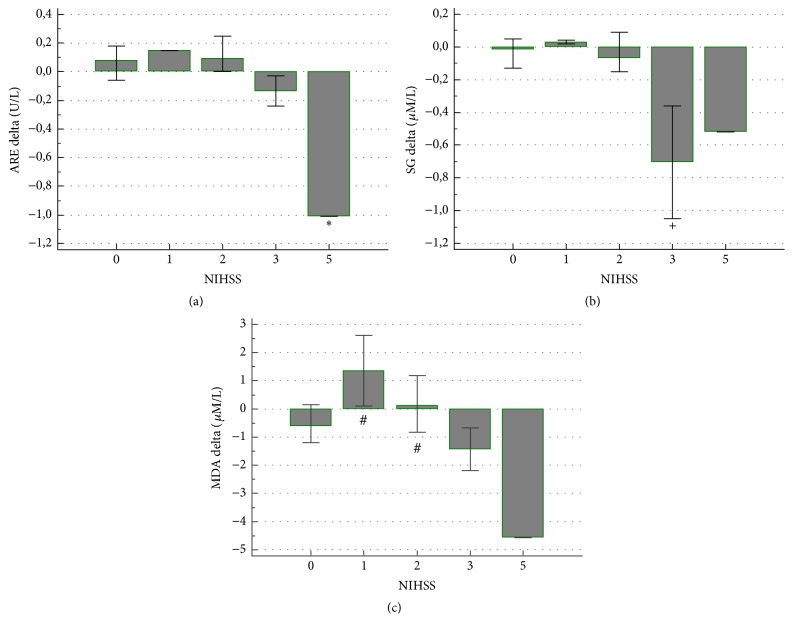
The association between the severity of neurological deficit and the change in ARE activity, SG, and MDA concentrations after CAS. ^*∗*^ *P* = 0.023, ^+^ *P* = 0.003, and ^#^ *P* = 0.013.

Patients with the history of TIA had more pronounced decrease in PON activity (−31.85; −114.05 to −12.55; median; interquartile range; *P* = 0.035) than those without stroke history or with ischemic stroke history (10.90; −23.40 to 10.00 and −8.30; −17.15 to 2.72, resp.). Patients without the history of cerebral ischemia had greater increase in CD (0.02; −0,05 to 0.32; median; interquartile range; *P* = 0.028) than the subjects with stroke or transient ischemic attack (TIA) history (−0.17; −0.39 to 0.06 and −0.19; −0.60 to −0.095, resp.). We have not identified the effects of other medical history data on the remaining analyzed parameters. Neurological deficit, as such, before CAS did not influence the change in PON and ARE activities, SG, CD, and MDA concentrations, but the severity of symptoms and disability played a role. The decrease in ARE activity after CAS was greater (*P* = 0.023) in the patient (one case) with highest NIHSS score comparing to the other subjects (Figure 2(a)), and, similarly, the decrease in SG (*P* = 0.003) was greater in patients with more severe neurological deficit measured with NIHSS (Figure 2(b)). MDA concentration increased (*P* = 0.013) in patients with mild NIHSS score comparing to more pronounced neurological deficit (Figure 2(c)).

The decrease in ARE activity was the greatest (*P* = 0.014) in patients with the highest Rankin score comparing with lower Rankin score or not disabled subjects. Similarly, the lowering in ARE activity was more pronounced (*P* = 0.022) in the patient with limited activity of daily living expressed in lower Barthel index score comparing to more independent subjects. The clinimetric measures did not affect other analyzed parameters.

The severity of carotid artery stenosis influenced only enzymatic antioxidant defense markers before CAS. We have found positive correlation with PON (*r*_*S*_ = 0.642, *P* = 0.003), ARE (*r*_*S*_ = −0.535, *P* = 0.0182), and PON/ARE (*r*_*S*_ = 0.621, *P* = 0.0045). The only parameter indicating the change in antioxidant defense after CAS that correlated with the degree of carotid artery stenosis was PON/ARE ratio (*r*_*S*_ = −0.507, *P* = 0.0268).

In logistic regression analysis of the factors affecting increase or decrease of studied parameters in the models including age, history of diabetes, hypertension, coronary heart disease, endovascular procedure, stroke, or TIA we have found the following:

(i) History of diabetes was associated with the increase in PON activity after CAS (*b* = 1.84; OR = 6.50; 95% CI: 1.05–40.24; *P* = 0.0441).
(ii) History of stroke was associated with decrease in CD after CAS (*b* = − 2,81; OR = 0.06; 95% CI: 0.007–0.54; *P* = 0.0124).
(iii) Age independently influenced the increase in SG (*b* = 0.16; OR = 1.21; 95% CI: 1.03–1.41; *P* = 0.0202).

## 4. Discussion

In our study on the effect of carotid artery stenting on antioxidant capacity and oxidative stress in patients with carotid artery stenosis we have observed acute effect of endovascular procedure on both enzymatic and nonenzymatic antioxidant capacity.

In the present study carotid artery stenting caused over one day the decrease in PON activity, which was more pronounced in patients with the history of TIA, but diabetics tended to present increased PON activity. Acute cerebral ischemia was associated with the decrease in PON activity [30]. Ferretti et al. observed that disturbed PON activity was associated with severe neurological deficit [31]. In our previous study we have found the effects of impaired PON and ARE activities on ischemic stroke outcome during short- and long-term observation [16]. Moreover, we have identified the predictive value of PON to ARE ratio [16]. We are not aware of the studies which investigated the effects of TIA on PON activities. However, endovascular procedures, for example, coronary angioplasty, influenced the enzymatic antioxidant capacities. In experimental study oxidative stress potentiated the injury of vascular smooth muscle cells after artery ballooning. Such an effect was associated with decreased enzymatic antioxidant defense represented by decreased glutathione peroxidase activity [32]. Stent implantation in experimental animals caused more severe injury than coronary artery balloon angioplasty and led to increased expression of C-reactive protein and oxidized phosphatidylcholine in the neointima [33]. The observation that paraoxonase 2-deficient apoE^−/−^ mice develop enhanced mitochondrial oxidative stress and exacerbated atherosclerosis [34] supports the evidence for a role of disturbed PON activity in vascular injury. In human studies, percutaneous transluminal coronary angioplasty did not change enzymatic antioxidant defense, while decreased enzymatic activities of erythrocytes' superoxide dismutase and catalase were caused by acute myocardial infarct [35].

The modification of PON activity early after CAS may be caused by several factors affecting the enzyme protein. The decrease of PON activity observed in our study can be a phenomenon related to acute phase response. Such an effect has been already reported [36]. Moreover, PON activity can be modified by immune-mediated mechanisms [37].

In our studied cohort we have not observed the effects of comorbidities analyzed as simple correlations with analyzed biomarkers. However, in logistic regression the diabetes was an independent factor associated with increase in PON activity after CAS. PON activity is known to be modified by a number of diseases including diabetes, cardiovascular diseases, liver cirrhosis, and renal diseases [38]. PON activity in diabetic patients reported by many studies was increased [39–41] comparing to healthy controls. The enhancement of PON activity after CAS in diabetic patients observed in the present study can be explained by nearly normal HDL-C concentrations used to reflect HDL antiatherogenic properties in clinical practice. PON constitutes principal antioxidant activity of HDL; thus it is highly correlated with the concentration of the HDL cholesterol. Our diabetic patients have sufficient antioxidant capacity, which may be additionally stimulated by phospholipids levels. Phosphatidylcholine was identified as major circulating phospholipid and stimulator of PON activity [42]. In experimental diabetes the production of phosphatidylcholine was found to be upregulated in kidneys [43], but it is also very susceptible to oxidation [44]. In diabetic patients phosphatidylcholine contains less polyunsaturated fatty acids; thus it is modified qualitatively [45].

In the present study we observed that the same endovascular procedure which decreased PON activity caused upregulation of ARE activity. However, in more disabled patients ARE activity was decreased. PON and ARE differ in their function and significance. PON is associated with paraoxon hydrolysis and represents enzymatic activity, but phenylacetate hydrolysis (ARE) reflects the mass of enzyme protein [15, 46, 47]. The major factors that can differentially modify PON and ARE activity are lysophospholipids. Palmitoyl-lysophosphatidylglycerol and lysophosphatidylinositol downregulate PON and upregulate ARE [48]. Carotid artery stenting can cause the release or production of lysolipids, which are overexpressed in carotid atherosclerotic plaques [49] and activated platelets [50]. Such a hypothetical mechanism could produce the changes which we observed in our study. Moreover, in the enzyme complex the site containing free sulfhydryl groups and responsible for protection of LDL against oxidation is independent of sites of PON and ARE activity [50].

PON activity analyzed in serum represents high interindividual variability determined by polymorphisms in the coding region of PON1 gene and* Q192R* and PON1 −*107T* allele [51]. In the present study we have undertaken the analysis only at the level of enzyme activity. This is a limitation which makes it impossible to provide the evidence for effects of polymorphism on enzymatic activity.

In our study, we have also observed after CAS a decrease in SG level, which was more pronounced in patients with severe neurological deficit expressed as higher NIHSS score. Animal experimental study on the effects of artery ballooning and oxidative stress on the injury of vascular smooth muscle cells revealed decrease in blood glutathione concentration after the procedure [32]. In humans, plasma sulfhydryl groups concentrations decreased within 24 hours after ischemic stroke onset and correlated positively with interleukin-8 and negatively with chitotriosidase (a marker of macrophage activation) and matrix metalloprotease 2 [18]. The latter study shows that plasma sulfhydryl groups concentration can be modified by acute inflammatory/immune response; thus it can be the case after CAS. Furthermore, the decrease in SG concentration was more pronounced in patients with higher NIHSS score, which indicates greater disability. Surprisingly, in our patients age was associated with the increase in SG concentration after CAS. In general, antioxidant defense decreases with the age [52, 53]. Our observation could be explained by an increased homocysteine concentration, which is observed in Polish population and independently associated with the fatal outcome of cardiovascular diseases [54]. Homocysteine, which belongs to the sources of sulfhydryl groups, together with glutathione and cysteine may increase with age as a result of decreased activation of cystathionine synthase [55]. The accumulation of homocysteine may on one hand increase SG concentration, but on the other hand increase the risk of vascular complications.

In general, we have not noticed the effects of CAS on CD. However, in female patients it increased and in patients with the history of stroke CD concentration decreased after CAS. Concentration of CD can be increased during reperfusion [56] and their decrease was associated with the enhancement of neurological recovery after cerebral ischemia [57]. Coronary angioplasty induced increased CD production in great cardiac vein already 1 minute after procedure, but no gender differences were reported [58]. No data are available on CAS effects on CD concentrations.

We have observed increased MDA concentration only in females after CAS. Together with CD increase it shows that females are more susceptible to lipid peroxidation after CAS. However we have not found differences in LDL cholesterol, and triacylglycerols concentrations between females and males (data not shown). Moreover, MDA concentration increased in our patients with mild NIHSS score comparing to more pronounced neurological deficit. The stimulation of MDA production was noticed after coronary angioplasty and was reduced by metoprolol [59]. Percutaneous transluminal coronary angioplasty stimulated the production of isoprostane F2a-III and isoprostane F2a-VI, which are considered as markers of lipid peroxidation and oxidative stress [60].

## 5. Conclusions

Carotid artery stenting influences both enzymatic (differently PON and ARE activity) and nonenzymatic antioxidant defense. Females are more susceptible to lipid peroxidation after CAS. PON/ARE ratio after CAS correlates with the degree of carotid artery stenosis. The changes in ARE activity, SG, and MDA concentrations are associated with the severity of neurological deficit and disability.

## Figures and Tables

**Table 1:** Demographic and clinical data, medical history, and comorbidities in studied cohort of patients.

Variable	Total *N* = 43 (100%)	Females *N* = 12 (28%)	Males *N* = 31 (72%)	*P*
Age [years] (mean ± SD)	64 ± 9	65 ± 10	63 ± 8	*P* = 0.5044
Carotid stenosis [%] (mean ± SD)	82 ± 10	82 ± 8	82 ± 10	*P* = 0.8830
NIHSS (median; interquartile range)	0; 0–2	0.5; 0–2.5	0; 0-1	*P* = 0.1902
Modified Rankin scale (median; interquartile range)	0.0; 0.0–1.0	1.0; 0.5–1.0	0.0; 0.0–1.0	*P* = 0.0919
Barthel index (median; interquartile range)	100; 95–100	97.5; 95–100	100; 95–100	*P* = 0.4423
Coronary heart disease	47%	38%	50%	*P* = 0.5603
Hypertension	74%	75%	73%	*P* = 0.9125
Diabetes	30%	38%	28%	*P* = 0.6182
Atrial fibrillation	6%	12%	4%	*P* = 0.3556
Stroke/transient ischemic attack (TIA)	33%/12%	40%/0%	28%/16%	*P* = 0.2245/*P* = 0.4299
Myocardial infarct	33%	25%	36%	*P* = 0.5905
History of endovascular procedure	30%	43%	27%	*P* = 0.3919

**Table 2:** Results of routine laboratory tests.

Parameter	Before CAS
Total cholesterol [mg/dL]	152 (135–173)
HDL cholesterol [mg/dL]	44 (38–51)
LDL cholesterol [mg/dL]	74 (70–110)
TAG [mg/dL]	130 (91–138)
Glucose [mg/dL]	96 ± 14
WBC [G/L]	8 ± 1
PLT [G/L]	234 ± 66

Data are presented as median and interquartile range (in parenthesis) or mean ± standard deviation.

**Table 3:** PON and ARE activities, PON/ARE ratio, SG, MDA, and CD concentrations in serum before and after CAS.

Parameter	Before CAS	After CAS	*P*
PON [U/L]	55.3 (40.2–161.8)	52.0 (33.2–132.9)	*P* = 0.0032
ARE [U/L]	1.42 ± 0.50	1.55 ± 0.49	*P* = 0.0058
PON/ARE	43.9 (21.3–134.9)	43.0 (19.7–98.5)	*P* = 0.3182
SG [*μ*M/L]	0.44 (0.36–0.47)	0.39 (0.32–0.45)	*P* = 0.0267
MDA [nM/L]	6.78 ± 1.47	6.37 ± 1.66	*P* = 0.0995
CD [*μ*M/L]	1.06 (0.83–1.33)	1.02 (0.83–1.38)	*P* = 0.7997

Data are presented as median and interquartile range (in parenthesis) or mean ± standard deviation.

**Table 4:** The differences (deltas) in PON and ARE activities, PON/ARE ratio, SG, MDA, and CD concentrations in serum of female and male patients. Delta = [the value after CAS] − [the value before CAS].

Parameter	Females	Males	*P*
Delta PON [U/L]	−15.0 (−22.6 to 2.9)	−11.7 (−35.6 to 2.98)	*P* = 0.8391
Delta ARE [U/L]	0.03 (−0.49 to 0.14)	0.12 (−0.04 to 0.21)	*P* = 0.1565
Delta PON/ARE	−9.65 (−45.08 to 1.09)	−12.84 (−53.24 to −1.95)	*P* = 0.5696
Delta SG [*μ*M/L]	−0.03 (−0.41 to 0.038)	−0.07 (−0.24 to 0.02)	*P* = 0.9544
Delta MDA [nM/L]	1.00 (1.00 to 2.00)	−0.82 (−1.51 to 0.22)	*P* = 0.0004
Delta CD [*μ*M/L]	1.00 (1.00 to 2.00)	−0.01 (−0.22 to 0.09)	*P* < 0.0001

Data are presented as median and interquartile range (in parenthesis) or mean ± standard deviation.
